# 

**Published:** 1892-12-17

**Authors:** 


### CONTENTS.

| | PAGE |
|---|---|
| The Madness of the Millionaire | 177 |
| Passing Topics.—The Guinea | 178 |
| Medical History from the Earliest Times. By E. T. Withington, M.A., M.B.Oxon. XXXVI.—The School of Salerno | 179 |
| The Attitude of Great Writers Towards Disease. XIX. Charles Lamb | 180 |
| Annotations.—Ophthalmia at Hanwell—Milk v. Milk Skimmed—Medical Watchmen and Cholera | 181 |
| Notes and News | 182 |
| Diet in Disease. By Mrs. Ernest Hart. X.—Digestion—Absorption—Excretion | 183 |
| Miss Ethel M. Warner and Mr. Lawson Tait | 192 |
| The Student of Medicine—Appointments—Vacancies—Pass Lists. | |
| The Hospital Clinic— | |
| Hospital for Sick Children, Great Ormond Street.—The Treatment of Rickets | 185 |
| Royal Infirmary, Edinburgh.—The Treatment of Some of the Complications and Sequelæ of Middle Ear Suppuration | 186 |
| Sussex County Hospital.—The Treatment of Acute Chorea | 187 |
| The Practitioners' Bookshelf.—The Human Body—A New Periodical—Letts' Diaries | 188 |
| New Drugs, Appliances, and Things Medical.—Electro-Medical Instruments (Schall)—Eucalyptus Oil | 188 |
| Books Received | 188 |
| The Institutional Workshop— | |
| Within the Hospitals.—Bradford Infirmary | 189 |
| Practical Departments.—Bedsteads (*continued*) | 191 |
| Hospital Worthies.—Charles Hamilton Fasson, M.D. | 191 |

### EXTRA SUPPLEMENT.—THE NURSING MIRROR.

| | |
|---|---|
| En Passant.—Lady Dufferin's Hospital, Agra—Lyme Regis—A New Nursing School—American Notes—"Long Expected"—The Scilly Isles—A Provident Nursing Scheme—Sectarianism—Fees and Co-operation—A Tribute | lxxxix |
| The Development of Children by Gymnastics.—I. Popular Notions | xc |
| The Royal British Nurses' Soiree | xci |
| Notes and Queries | xci |
| Working and Waiting | xci |
| Nurses in Germany | xcii |
| Everybody's Opinion.—Nurses and their Fees—Wanted, a Home for the Cure of Hysteria | xcii |
| Medico-Psychological Association | xcii |
| Appointments | xcii |
| Novelties for Nurses | xciii |
| Wants and Workers | xciii |
| For Reading to the Sick.—Melancholy | xciii |
| Some Australian Experiences.—Off to the Gold Fields | xciv |

